# Altered balance control in thoracic adolescent idiopathic scoliosis during obstructed gait

**DOI:** 10.1371/journal.pone.0228752

**Published:** 2020-02-06

**Authors:** Kuan-Wen Wu, Tung-Wu Lu, Wei-Chun Lee, Ya-Ting Ho, Ting-Chun Huang, Jyh-Horng Wang, Ting-Ming Wang

**Affiliations:** 1 Department of Biomedical Engineering, National Taiwan University, Taiwan, ROC; 2 Department of Orthopaedic Surgery, School of Medicine, National Taiwan University, Taiwan, ROC; 3 Department of Orthopaedic Surgery, National Taiwan University Hospital, Taiwan, ROC; 4 Department of Orthopaedic Surgery, Chang Gung Memorial Hospital, Taiwan, ROC; 5 Department of Orthopaedic Surgery, Chu-Tung Branch, National Taiwan University Hospital, Taiwan, ROC; University of Massachusetts Lowell, UNITED STATES

## Abstract

Adolescent idiopathic scoliosis (AIS) is the most common spinal deformity during adolescence, leading to altered postural control with compromised stability. To identify the effects of AIS on whole-body balance control during obstacle-crossing, 14 adolescents with Lenke 1 thoracic AIS and 14 healthy controls were compared in terms of the inclination angle (IA) of the body’s center of mass (COM) relative to the center of pressure (COP), the rate of change of IA (RCIA) and the jerk index of IA. Between-side comparisons were also performed for the AIS group. The patients showed less smooth COM-COP motion in the sagittal plane with significantly increased anterior RCIA and IA jerk index during crossing with either the concave side (p = 0.001) or the convex side (p = 0.001) leading when compared to healthy controls. In the frontal plane, the patients showed close-to-zero RCIA (p = 0.002) while crossing with the leading limb, with an increased IA magnitude (p = 0.039) only while crossing with the concave-side limb leading. The patients with Lenke 1 thoracic AIS were found to cross obstacles with altered, compromised COM-COP control in both sagittal and frontal planes when compared to healthy controls. The results suggest that the thoracic spinal deformity in Lenke 1 AIS affects the whole-body balance control during obstacle-crossing, which should be monitored for signs of increased risk of loss of balance in the management of such patient groups.

## Introduction

Adolescent idiopathic scoliosis (AIS) is the most common spinal deformity during adolescence, characterized by a lateral spinal curvature with an axial rotation and loss of sagittal kyphosis of the thoracic spine [1, 2]. This three-dimensional spinal deformity often alters the orientations of the head, trunk and pelvis in all anatomical planes [3–5], as well as resulting in vestibular and trunk muscle imbalance [6–8], and proprioceptive disorders [9], leading to postural control problems[10] with compromised postural stability [10, 11].

Poor body segmental alignment as a result of AIS has been associated with increased lateral displacement of the body’s center of mass (COM) [12], affecting the body’s dynamic balance during walking. The AIS has also been shown to affect gait mechanics, including temporal-distance parameters, ground reaction forces and kinematics of the lower limb joints [8, 13–15]. Such kinematic changes are thought to be a compensatory mechanism for maintaining whole-body balance during gait [16–18].

Compared to unobstructed gait, negotiating obstacles during walking places greater neuromechanical demands on the locomotor system, and has been identified as an environmental risk factor for falling [19]. A successful obstacle-crossing task requires precise control of the end-point of the crossing limb while maintaining whole-body balance through highly coordinated control of the motions of the trunk and the pelvis-leg segments. With the upper body accounting for 60% of the body mass, the asymmetrical AIS spinal deformity alters the shape and mass distribution of the trunk, and thus its center of mass position [20], affecting the coordinated control of the motions of the trunk and other body segments during obstacle-crossing. Depending on which limb is leading, i.e., the limb on the convex or concave side, the control of the motions of the body segments can be different with different balance strategies, especially when AIS occurs with impaired proprioception [9].

The body’s balance control during obstacle-crossing has been quantified by the motion of the body’s COM relative to the center of pressure (COP) in terms of the COM-COP inclination angle (IA), namely the angle formed by the vertical line and the line connecting the COP and COM, and the rate of change of IA (RCIA) [21, 22]. During walking and obstacle-crossing, the COM can be outside the constantly changing and moving base of support (BOS) and far away from the COP without loss of balance as long as the COM is controlled at an appropriate velocity [23]. This is in contrast to static conditions such as quiet standing during which a person is thought to be in balance as long as the downward vertical projection of the COM is kept within the BOS and mostly close to the COP. While the horizontal separation of the COM and COP has previously been used to study balance control during activities, it does not consider the influence of the body height or leg length, nor the velocity of the COM relative to the COP. The IA overcomes this limitation by combining both the information of the horizontal separation of the COM-COP, as well as the height of the COM, thus better enabling comparisons between subjects of different statures [22, 24], as well as the study of activities where horizontal COM-COP separation may not be appropriate, such as slope walking [25]. The RCIA further includes the information of the horizontal and vertical components of the velocity of the COM relative to the COP, and together with IA provides a more complete description of the control of the COM relative to the COP. Generally, the further the COM diverges from the COP (i.e., greater IA) the more difficult it becomes, and more effort (e.g., joint torque) is needed to achieve a rate of change of IA (i.e., RCIA to reduce IA) appropriate for dynamic balance [23]. For example, in the sagittal plane during the first half of single-limb support (SLS) the body’s COM is initially outside and posterior to the BOS, i.e., the foot of the stance limb, and is moving towards the BOS with an anterior RCIA appropriate for the IA. During the second half of SLS the body’s COM is initially inside the BOS and is moving forward and away from the BOS until the swing foot makes contact with the ground to arrest the COM. Thus, for a smooth transition to the double-limb support phase, a decreasing anterior RCIA, changing to a posterior RCIA before the swing foot contact, is needed [21]. The IA and RCIA together have been useful for studying the differences in the balance control strategies between different subject populations during dynamic activities [24, 26, 27], such as distinguishing healthy elderly from those who had experienced falls within a 2-year period [22], and for investigating the dynamic balance control in patients with knee osteoarthritis [27].

Apart from the above-mentioned experimental data-based indices, there are methods that were developed to predict stability margins or regions [28, 29] or to predict how foot placement can be used to control gait stability [30]. For example, the extrapolated center of mass (XcoM) is an imaginary point combining the position and velocity of the COM based on an inverted pendulum model, which enables the definition of the region of dynamic stability [31, 32]. While these methods are useful for studying factors influencing dynamic stability during level walking, such as walking speed and foot placement, they may not be suitable for activities or studies in which the assumptions and conditions involved do not hold. In contrast, the IA and RCIA are defined by the measured COM and COP motions without involving mathematical models and their associated assumptions that are not met in the current study. Therefore, the IA and RCIA variables are considered more appropriate for the current study.

Patients with AIS have been shown to change the motions of the trunk and the pelvis-leg apparatus during obstacle-crossing as compared to level walking [33]. These changes are expected to affect the balance control of the body and increase the neuromechanical demand on the locomotor system. However, to the best knowledge of the authors, no study has investigated the effects of AIS spinal deformity on balance control in terms of COM-COP motions during obstacle-crossing. Therefore, the purpose of this study was to identify the influence of spinal deformity on the whole-body balance control during obstacle-crossing in terms of COM-COP motions in patients with Lenke 1 thoracic AIS. It was hypothesized that patients with AIS would cross obstacles with altered COM-COP control when compared to healthy controls, and that the changes would be greater when crossing with the limb on the concave side leading than when crossing with the convex side leading.

## Methods

This study was approved by the Research Ethics Committee of National Taiwan University Hospital (Permit Number: 201306013RINB). All methods were carried out in accordance with relevant guidelines and regulations. Fourteen adolescents with right thoracic AIS (AIS group; age: 14.0±1.8 years, height: 154.8±4.7 cm, mass: 42.0±7.5 kg) were recruited from the university hospital between July 2013 and May 2016. All participants gave written informed consents signed by both them and their legal guardians. All the patients were determined radiographically to have a Lenke 1 thoracic curve[34] with Cobb angles of 59.9±18.9° and kyphosis angles of 28.2±8.8°. The patients had normal lower limb muscle strength, with corrected vision and right leg dominance without limitations in performing daily or sports activities. The only treatment was wearing a brace during the daytime. All indicated studies were performed without the brace. Participants were excluded if they had leg length discrepancies greater than 1 cm as determined by scanogram, or other musculoskeletal diseases that would affect their gait performance. Fourteen healthy adolescents (Control group; age: 14.4±2.0 years, height: 158.4±6.2 cm, body mass: 48.6±8.9 kg) were selected to match with the AIS group for sex, age and BMI. An *a priori* power analysis based on pilot results of sagittal and frontal IA and RCIA from four patients with AIS and four healthy subjects using GPOWER 3 [35] determined that a projected sample size of ten subjects for each group would be enough for a power of 0.8 and an effect size of 0.9 at a significance level of 0.05. Thus, 14 subjects for each, namely the AIS and the control group, were adequate for achieving the main objectives of this observational, cross-sectional study.

In a university hospital gait laboratory, each subject walked at a self-selected pace on a 10-meter walkway and crossed an obstacle composed of a 1.5 m long tube with a diameter of 1.5 cm placed horizontally across a height-adjustable metal frame [36]. Three obstacle heights (i.e., 10%, 20% and 30% of leg length) were tested with each leg leading while the motions of the body segments were monitored by an 8-camera motion analysis system (Vicon MX T-40) at 200 Hz using 39 infrared-retroreflective skin markers [37]. The ground reaction forces were also measured simultaneously at 2000 Hz using two forceplates (OR6-7, AMTI) placed on either side of the obstacle in the middle of the walkway. With the current forceplate setup, the crossing cycle was defined from the toe-off of the leading limb before crossing to the toe-off of the leading limb after crossing. The ground reaction force data were used to determine the toe-off of the leading limb, heel-strike of the leading limb, and toe-off of the contralateral limb. The marker data were used to determine the instants when the leading toe was above the obstacle, and when the trailing toe was above the obstacle as well as the heel-strike of the contralateral limb. Data for three complete crossing cycles for each lower limb leading were obtained for each obstacle condition. For the AIS group, the crossing cycle with the limb on the convex side leading was denoted AIS-V and the cycle with the limb on the concave side leading was denoted AIS-A. For the crossing limb conditions (AIS-V vs. AIS-A) a counterbalanced measures design was used, while the sequence of the obstacle conditions was decided by a random number table.

The body’s COM position was calculated as the weighted sum of the positions of the COMs of all the body segments using the marker data and segmental inertial properties. Subject-specific body segmental inertial properties were obtained using an optimization-based method, which has been shown to reduce errors in the calculated center of mass motions when compared to commonly used prediction methods [38]. The COP position was calculated using forces and moments measured by the forceplates. The COM-COP inclination angles (IA) in the sagittal and frontal planes were then calculated as follows:

$$\overset{⃑}{t}=\left(\overset{⃑}{Z}\times \frac{{\overset{⃑}{P}}_{COM-COP}}{|{\overset{⃑}{P}}_{COM-COP}|}\right)$$ (1)

$$Sagittal\;IA=\sin^{-1}\left(t_{Y}\right)$$ (2)

$$Frontal\;IA=\left\{ \begin{array}{c} {-\sin}^{-1}(t_{X}),\;for\;the\;right\;limb\\ \sin^{-1}\left(t_{X}\right),\;for\;the\;left\;limb \end{array}\right.$$ (3)

where ${\overset{⃑}{P}}_{COM-COP}$ was the vector pointing from the COP to the COM, $\overset{⃑}{Z}$ was the unit vector of the vertical and $\overset{⃑}{X}$ was the unit vector pointing in the direction of progression. With the current forceplate setup, the IAs were calculated from the beginning of swing phase of the leading limb to the subsequent contralateral heel-strike. The RCIA were calculated by smoothing and differentiating the IA trajectories using the GCVSPL method[39]. For the leading limb, positive sagittal and frontal IA indicate that the COM is anterior to and away from the COP toward the contralateral limb, respectively. The greater the IA, the greater the COM-COP vector deviates from the vertical and the greater the effort needed to bring the COM back to be above the COP unless accompanied by an appropriate RCIA to reduce IA, corresponding to the position and velocity control of the COM described by Pai *et al*. [23]. To quantify the smoothness of the balance control, the jerk index of the COM-COP motion was calculated for contralateral single-limb support (SLS) and double-limb support (DLS) during crossing using the third derivatives of the IA trajectories as follows [40–43].

$$Jerk\;Index=\int_{t_{i}}^{t_{f}}{\left(IA\prime \prime \prime \right)}^{2}\;dt$$ (4)

where *t*_*i*_ and *t*_*f*_ are, respectively, the beginning and end of the sub-phase considered.

Crossing speed was calculated as the ratio of the distance traveled by mid-ASISs in the walking direction and the time spent from leading toe-off immediately before crossing to trailing heel-strike immediately after crossing. Toe-clearance for both the leading and trailing limb was calculated as the vertical distance between the toe marker of the swing limb and the obstacle when the swing toe was directly above the obstacle. The trailing toe-obstacle horizontal distance was defined as the horizontal distance between the obstacle and the toe marker of the trailing limb during stance immediately before stepping over the obstacle. The leading heel-obstacle horizontal distance was defined as the horizontal distance between the obstacle and the heel marker of the leading limb during stance immediately after stepping over the obstacle. The leading toe-obstacle horizontal distance was defined as the horizontal distance between the obstacle and the toe marker of the leading limb during stance immediately before stepping over the obstacle [24].

For statistical analysis, the values of the IA and RCIA when the leading and trailing toes were directly above the obstacle were obtained. The range of motion and mean of IA, and the peak RCIA during DLS as well as the mean of IA during SLS of the trailing limb were also obtained. For each calculated variable, data from three trials were averaged for AIS-V and for AIS-A, while those from both sides (i.e., six trials) were averaged for Control. All calculated variables were determined to be normally distributed by a Shapiro-Wilk test and the homogeneity of variance across groups was confirmed by the Levene's test. For between-group comparisons, a two-way mixed-design analysis of variance (ANOVA) was used to compare the differences in all the calculated variables with one between-subject factor (i.e., AIS-V vs. Control, and AIS-A vs. Control) and one within-subject factor (obstacle height). For between-side comparisons in AIS, a two-way repeated-measures ANOVA was used to compare the differences with one within-subject factor (AIS-V vs. AIS-A) and another separate within-subject factor (obstacle height). Whenever an obstacle height effect was found, a *post hoc* trend analysis was performed to determine the trend of the variable. A significance level of α = 0.05 was set for all test conditions. All statistical analyses were performed using SPSS version 20 (SPSS Inc., Chicago, IL, USA).

## Results

Compared to the healthy controls, no significant differences were found in the crossing speed (Table 1). The AIS group showed significantly increased leading heel-obstacle horizontal distance for all obstacle heights, both when crossing with the limb on the concave side leading (AIS-A) and when crossing with the convex side leading (AIS-V), while the trailing toe-obstacle horizontal distances were not significantly different between groups. Double-limb support (DLS) time for both AIS-V and AIS-A was significantly decreased but only AIS-V showed significantly increased leading swing phase time when compared to the control group.

**Table 1:** Means (standard deviations, SD) of the end-point variables in the patients with adolescent idiopathic scoliosis and healthy controls when crossing obstacles of heights of 10%, 20% and 30% of the subjects’ leg length.

Variables	Obstacle Height (%LL)	AIS-V	AIS-A	Control	P_V_	P_H_	P_S_
					P_A_		
Crossing speed(mm/s)	10	1013.9 (139.2)	1009.4 (131.6)	951.7 (82.2)		0.000↓	0.976
	20	945.7 (109.3)	952.2 (127.8)	897.9 (84.4)	0.215		
	30	900.2 (106.5)	897.2 (126.7)	865.8 (98.9)	0.248		
Leading toe-clearance(%LL)	10	15.9 (4.2)	15.9 (4.8)	18.5 (3.0)		0.462	0.271
	20	15.7 (4.8)	15.2 (4.4)	17.7(3.0)	0.141		
	30	15.3 (3.8)	14.1 (2.4)	16.4 (2.2)	0.051		
Trailing toe-clearance(%LL)	10	12.6 (3.6)	13.4 (4.7)	16.1 (3.3)		0.218	0.922
	20	13.6 (5.4)	12.9 (5.9)	16.6 (4.5)	0.040*		
	30	14.9 (4.7)	15.0 (5.0)	17.0 (3.2)	0.010*		
Trailing toe-obstacle distance(%LL)	10	21.2 (4.4)	20.0 (2.8)	22.6 (2.0)		0.000↑	0.130
	20	26.0 (2.7)	25.3 (2.9)	26.9 (1.9)	0.159		
	30	33.5 (2.5)	33.3 (1.9)	35.1 (1.2)	0.095		
Leading heel-obstacle distance(%LL)	10	24.3 (4.1)	23.2 (4.6)	19.4 (4.0)		0.000↑	0.484
	20	25.4 (3.3)	26.6 (4.3)	22.4 (3.3)	0.014*		
	30	29.7 (3.1)	29.5 (3.9)	27.7 (3.2)	0.046*		
Leading toe-obstacle distance(%LL)	10	74.2 (7.4)	73.7 (4.5)	80.9 (5.3)		0.002	0.859
	20	75.3 (4.4)	75.0 (4.5)	81.9 (5.9)	0.001*		
	30	77.4 (4.7)	77.8 (4.0)	85.6 (5.2)	0.000*		
Double-limb support time (%CC)	10	10.9 (1.1)	11.0 (1.4)	12.9 (0.8)		0.012↓	0.505
	20	10.9 (1.3)	11.2 (1.4)	12.3 (0.9)	0.000*		
	30	10.2 (1.4)	10.4 (1.4)	11.9 (0.9)	0.001*		
Contra single-limb support (%CC)	10	41.1 (1.1)	40.3 (2.2)	39.9 (1.4)		0.004↑	0.175
	20	41.8 (1.6)	41.3 (2.1)	40.8 (1.2)	0.023*		
	30	42.4 (1.6)	41.7 (2.6)	41.6 (1.1)	0.601		

Abbreviations: %LL, % leg length; %CC, % crossing cycle; AIS-V, crossing cycle with the convex-side limb leading; AIS-A, crossing cycle with the concave-side limb leading; P_H_ = *p*-value for obstacle height; P_V_ = AIS-V vs. Control; P_A_ = AIS-A vs. Control; P_S_ = AIS-A vs. AIS-V; Contra single-limb support, contralateral single-limb support time.

*: AIS-V or AIS-A significantly different from Control

↑ indicates linearly increasing trend.

↓ indicates linearly decreasing trend.

**Fig 1:**
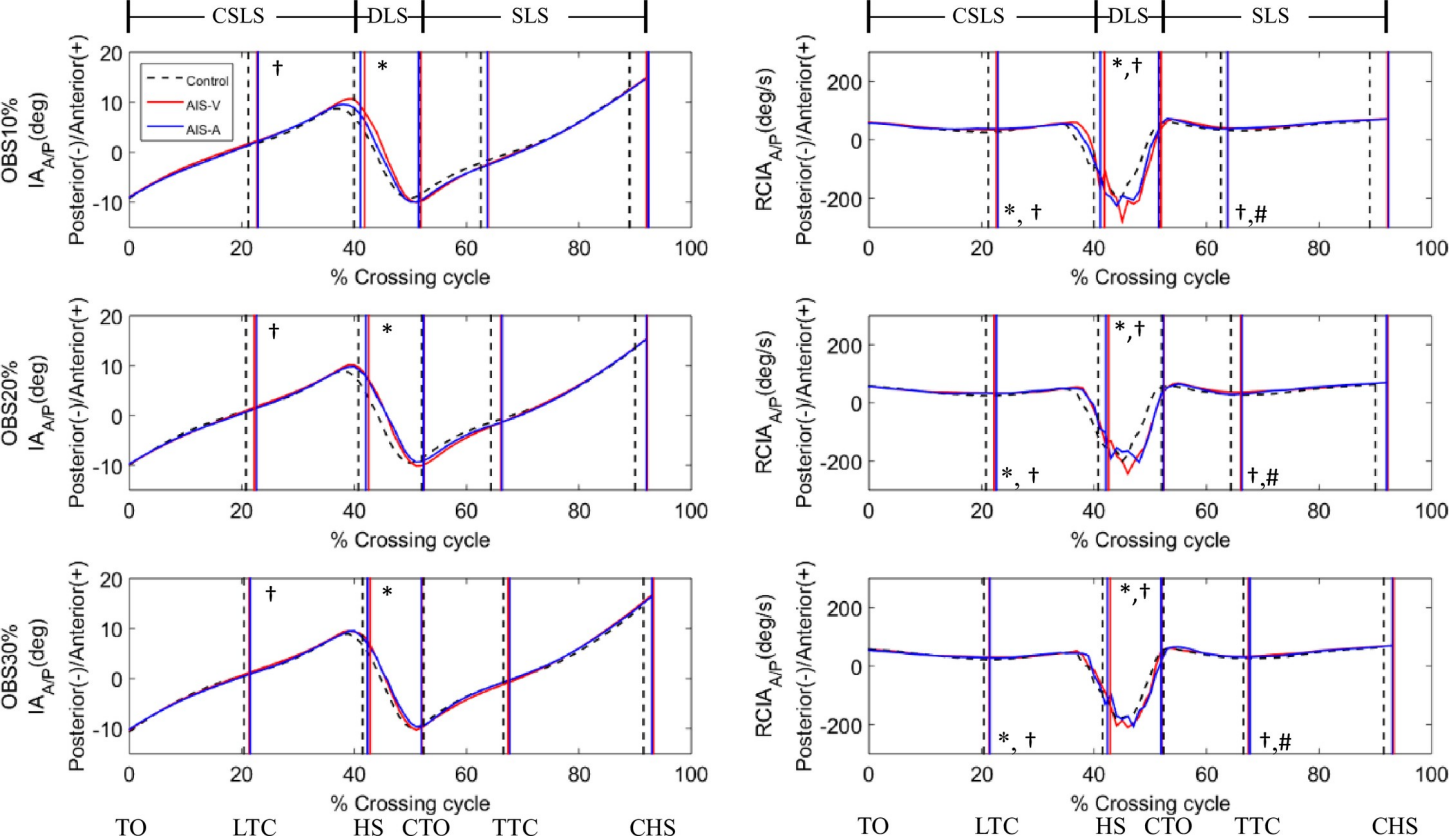
Ensemble-averaged curves of (A) COM-COP inclination angle (IA), and (B) rate of change of IA (RCIA) in the sagittal plane over the crossing cycle when crossing obstacles of heights of 10%, 20% and 30% the leg length (LL) for convex side, concave side and controls, drawn with data points at an interval of 1% crossing cycle. The anterior/posterior (A/P) position of the COM and COP were described parallel to the direction of progression, a zero value being the position of toe-off and a positive value being anterior to that position. (TO: toe-off of the leading limb; LTC: leading toe above the obstacle; HS: heel-strike of the leading limb; CTO: toe-off of the contralateral limb; TTC: trailing toe above the obstacle; CHS: heel-strike of the contralateral limb; SLS: single-limb support of the leading limb; CSLS: contralateral single-limb support; DLS: double-limb support; *: AIS-A significantly different from Control; †: AIS-V significantly different from Control; #: AIS-A significantly different from AIS-V).

**Fig 2:**
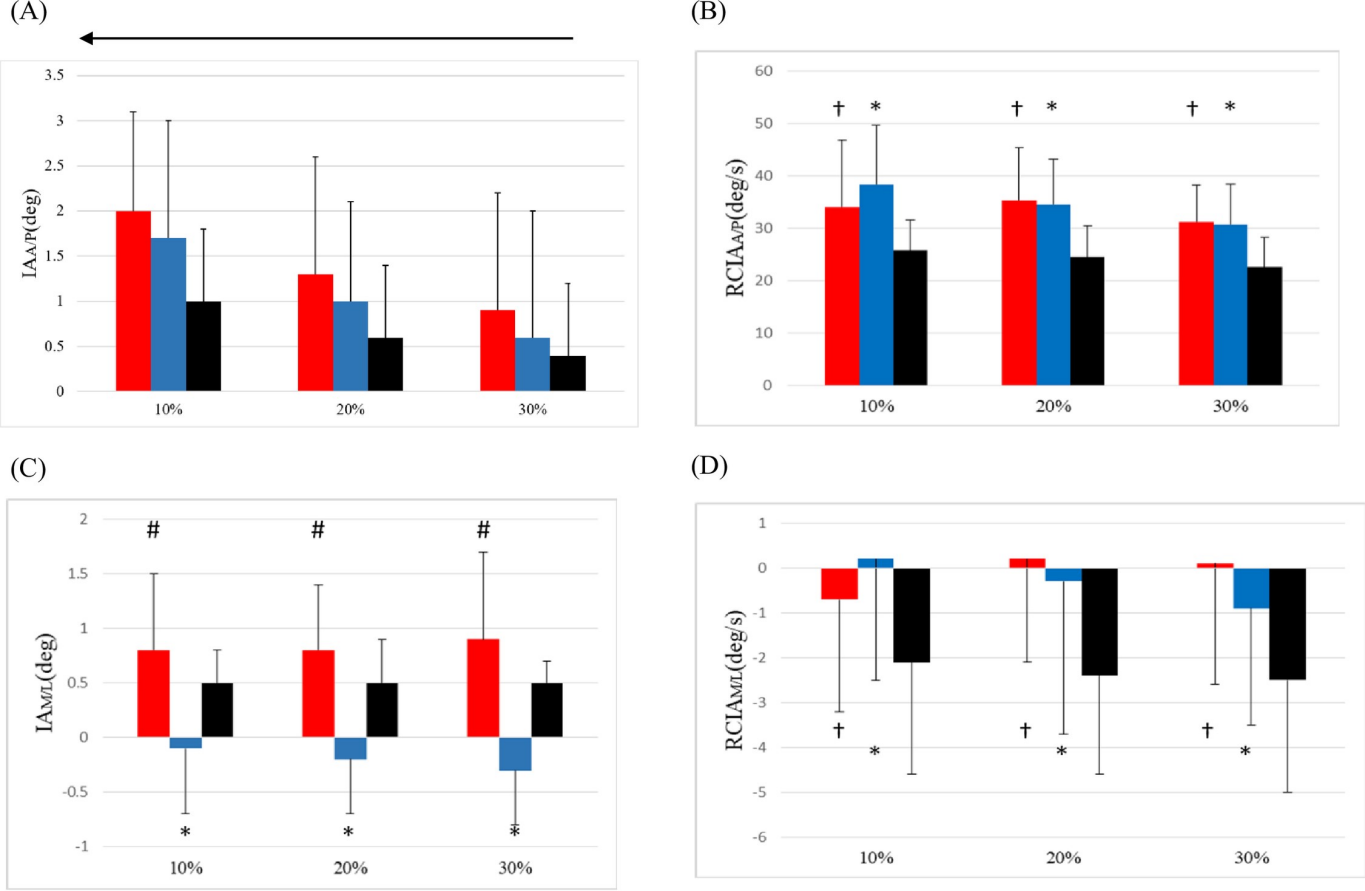
Comparisons of the mean IA and RCIA between groups when the leading toe was above the obstacle with the corresponding standard deviations shown as error bars: (A) sagittal IA, (B) sagittal RCIA, (C) frontal IA, and (D) frontal RCIA. *: AIS-A significantly different from Control. †: AIS-V significantly different from Control. #: AIS-A significantly different from AIS-V. With increasing obstacle height, a left arrow indicates a linearly decreasing trend (red bar: AIS-V; blue bar: AIS-A; black bar: Control).

In the sagittal plane, averaged IA values over the leading swing phase and the ranges of IA during DLS were increased when the limb on the convex side was leading (Fig 1 and Table 2). Compared to Control, no significant differences were found in any of the IA-related variables during AIS-A, except for the increased averaged values over the DLS. Both AIS-V and AIS-A showed significantly increased anterior RCIA when the leading toe was above the obstacle (Fig 2) but only AIS-V showed increased anterior RCIA when the trailing toe was above the obstacle. Both AIS-V and AIS-A showed significantly decreased mean and peak values of RCIA over DLS (Table 3). When compared to Control, both AIS-V and AIS-A showed an increased jerk index of IA over DLS and CSLS (Table 4).

**Table 2:** Means (standard deviations, SD) of the sagittal and frontal COM-COP inclination angles (°) in the patients with adolescent idiopathic scoliosis and healthy controls when crossing obstacles of heights of 10%, 20% and 30% of subjects’ leg length.

	Obstacle Height (%LL)	AIS-V	AIS-A	Control	P_V_	P_H_	P_S_
					P_A_		
Sagittal plane							
Leading toe above obstacle	10	2.0 (1.1)	1.7 (1.3)	1.0 (0.8)		0.002↓	0.325	
	20	1.3 (1.3)	1.0 (1.1)	0.6 (0.8)	0.057			
	30	0.9 (1.3)	0.6 (1.4)	0.4 (0.9)	0.249			
Trailing toe above obstacle	10	-2.9 (2.2)	-3.0 (2.1)	-2.5 (1.3)		0.000	0.405	
	20	-2.0 (1.7)	-1.6 (2.6)	-1.8 (0.9)	0.718			
	30	-1.2 (2.2)	-0.7 (2.6)	-1.1 (0.8)	0.939			
Mean DLS	10	-1.1 (1.1)	-1.5 (1.5)	-1.9 (0.7)		0.054	0.194	
	20	-1.4 (1.5)	-1.0 (1.7)	-2.3 (0.8)	0.103			
	30	-2.0 (1.7)	-1.3 (1.4)	-2.2 (0.7)	0.048*			
Mean CSLS	10	1.6 (0.9)	1.1 (0.9)	0.7 (0.7)		0.019	0.082	
	20	1.0 (1.0)	0.7 (1.0)	0.4 (0.8)	0.046*			
	30	0.9 (1.2)	0.6 (1.1)	0.3 (0.7)	0.268			
ROM DLS	10	22.3 (2.1)	21.7 (2.1)	20.2 (1.6)		0.473	0.040	
	20	22.3 (2.0)	21.2 (2.1)	20.5 (1.3)	0.012*			
	30	22.2 (2.4)	21.5 (2.3)	20.9 (1.6)	0.157			
Frontal plane							
Leading toe above obstacle	10	-2.1 (0.8)	-3.0 (0.9)	-2.5 (0.3)		0.020	0.000^†^	
	20	-2.0 (0.6)	-2.8 (0.7)	-2.2 (0.5)	0.116			
	30	-2.0 (0.5)	-2.5 (0.6)	-2.2 (0.4)	0.039*			
Trailing toe above obstacle	10	3.2 (1.0)	2.4 (1.1)	2.8 (0.5)		0.570	0.020^†^	
	20	3.2 (0.8)	2.3 (0.9)	2.7 (0.6)	0.046*			
	30	3.3 (0.8)	2.1 (1.0)	2.8 (0.4)	0.087			
Mean DLS	10	0.8 (0.7)	-0.1 (0.6)	0.5 (0.3)		0.320	0.002^†^	
	20	0.8 (0.6)	-0.2 (0.5)	0.5 (0.4)	0.100			
	30	0.9 (0.8)	-0.3 (0.5)	0.5 (0.2)	0.000*			
Mean CSLS	10	-2.2 (0.7)	-3.1 (0.7)	-2.7 (0.3)		0.111	0.000^†^	
	20	-2.2 (0.6)	-3.0 (0.7)	-2.5 (0.6)	0.056			
	30	-2.2 (0.5)	-2.8 (0.7)	-2.5 (0.4)	0.062			
ROM DLS	10	6.6 (1.5)	6.7 (2.0)	6.8 (1.0)		0.963	0.865	
	20	6.6 (1.4)	6.6 (1.6)	6.6 (1.4)	0.604			
	30	6.8 (1.4)	6.4 (1.8)	7.2 (1.0)	0.565			

Abbreviations: %LL, % leg length; AIS-V, crossing cycle with the convex-side limb leading; AIS-A, crossing cycle with the concave-side limb leading; P_H_ = *p*-value for obstacle height; P_V_ = AIS-V vs. Control; P_A_ = AIS-A vs. Control; P_S_ = AIS-A vs. AIS-V; Mean DLS, mean values during double-limb support; Mean CSLS, mean values during contralateral single-limb support; ROM DLS, range of IA during double-limb support.

*AIS-V or AIS-A significantly different from Control.

^†^ significantly different between sides.

↑ indicates linearly increasing trend.

↓ indicates linearly decreasing trend.

**Table 3:** Means (standard deviations, SD) of the sagittal and frontal rates of change of COM-COP inclination angles (°/s) in the patients with adolescent idiopathic scoliosis and healthy controls when crossing obstacles of heights of 10%, 20% and 30% of subjects’ leg length.

	Obstacle Height (%LL)	AIS-V	AIS-A	Control	P_V_	P_H_	P_S_
					P_A_		
Sagittal plane								
Leading toe above obstacle	10	34.0 (12.8)	38.3 (11.4)	25.7 (5.9)		0.148	0.408
	20	35.3 (10.0)	34.5 (8.7)	24.4 (6.0)	0.001*		
	30	31.2 (7.0)	30.7 (7.6)	22.5 (5.7)	0.001*		
Trailing toe above obstacle	10	42.7 (11.4)	38.0 (13.7)	34.8 (6.3)		0.046	0.043
	20	39.3 (12.5)	31.8 (9.7)	30.9 (6.6)	0.025*		
	30	33.4 (12.4)	31.3 (12.1)	27.6 (9.7)	0.426		
Mean DLS	10	-165.8 (35.8)	-158.0 (50.9)	-112.0 (15.9)		0.051	0.314
	20	-155.4 (37.6)	-143.3 (43.8)	-108.7 (18.0)	0.000*		
	30	-154.0 (53.9)	-153.0 (44.9)	-107.2 (24.0)	0.002*		
Mean CSLS	10	38.3 (4.9)	38.2 (5.9)	34.4 (4.4)		0.000	0.226
	20	34.8 (5.2)	35.3 (5.9)	32.8 (4.3)	0.317		
	30	31.2 (5.9)	32.9 (5.1)	31.9 (4.3)	0.180		
Peak	10	-424.2 (164.6)	-349.9 (109.5)	-246.1 (44.4)		0.037	0.456
	20	-318.9 (79.5)	-330.8 (90.1)	-239.4 (51.6)	0.000*		
	30	-308.1 (97.3)	-324.0 (114.6)	-228.1 (54.6)	0.001*		
Frontal plane								
Leading toe above obstacle	10	-0.7 (2.5)	0.2 (2.7)	-2.1 (2.5)		0.720	0.608
	20	0.2 (2.3)	-0.3 (3.4)	-2.4 (2.2)	0.017*		
	30	0.1 (2.7)	-0.9 (2.6)	-2.5 (2.5)	0.038*		
Trailing toe above obstacle	10	-0.8 (2.4)	-2.0 (3.5)	0.2 (2.6)		0.947	0.367
	20	-1.3 (2.2)	-1.7 (3.1)	0.4 (2.1)	0.142		
	30	-1.7 (2.6)	-1.4 (2.6)	-1.1 (1.5)	0.075		
Mean DLS	10	47.1 (11.0)	47.6 (17.9)	39.3 (6.7)		0.297	0.736
	20	44.8 (9.6)	43.0 (12.2)	37.1 (10.5)	0.014*		
	30	46.5 (9.5)	43.7 (12.8)	39.0 (7.8)	0.124		
Mean CSLS	10	0.9 (1.6)	1.0 (1.5)	1.0 (1.3)		0.915	0.624
	20	1.0 (1.2)	1.0 (1.1)	0.5 (0.8)	0.245		
	30	1.2 (1.5)	0.6 (1.4)	0.2 (0.9)	0.414		
Peak	10	107.0 (49.2)	97.2 (40.2)	75.0 (14.8)		0.219	0.540
	20	87.8 (18.8)	91.3 (30.5)	69.0 (19.2)	0.005*		
	30	94.3 (23.0)	83.3 (28.3)	74.7 (16.4)	0.046*		

Abbreviations: %LL, % leg length; AIS-V, crossing cycle with the convex-side limb leading; AIS-A, crossing cycle with the concave-side limb leading; P_H_ = *p*-value for obstacle height; P_V_ = AIS-V vs. Control; P_A_ = AIS-A vs. Control; P_S_ = AIS-A vs. AIS-V; Mean DLS, mean values during double-limb support; Mean CSLS, mean values during contralateral single-limb support; peak, peak values during double-limb support.

*AIS-V or AIS-A significantly different from Control.

↑ indicates linearly increasing trend.

↓ indicates linearly decreasing trend

**Table 4:** Means (standard deviations, SD) of the jerk index (10^5^ °/s^3^) of the sagittal and frontal IA in the patients with adolescent idiopathic scoliosis and healthy controls when crossing obstacles of heights of 10%, 20% and 30% of subjects’ leg length.

	Obstacle Height (%LL)	AIS-V	AIS-A	Control	P_V_ P_A_	P_H_	P_S_
Sagittal plane								
DLS	10	52.1 (66.7)	28.6 (24.1)	19.9 (21.1)		0.162	0.195
	20	39.4 (30.8)	39.4 (33.7)	15.7 (81.6)	0.027*		
	30	54.8 (53.1)	47.8 (46.8)	18.0 (12.6)	0.027*		
CSLS	10	13.9 (19.7)	9.0 (9.7)	4.8 (3.2)		0.117	0.579
	20	9.7 (8.1)	12.3 (10.9)	5.0 (3.0)	0.026*		
	30	19.5 (22.1)	17.9 (26.9)	6.0 (4.4)	0.042*		
Frontal plane								
DLS	10	12.0 (11.2)	5.8 (5.2)	4.5 (4.8)		0.231	0.090
	20	10.4 (7.7)	7.9 (6.1)	3.9 (1.9)	0.022*		
	30	15.3 (18.6)	11.2 (9.5)	5.6 (3.4)	0.051		
CSLS	10	2.5 (2.6)	2.0 (2.5)	1.2 (0.7)		0.140	0.443
	20	2.2 (2.2)	2.3 (2.0)	1.0 (0.5)	0.041*		
	30	4.5 (5.7)	3.5 (4.2)	1.7 (1.6)	0.061		

Abbreviations: %LL, % leg length; AIS-V, crossing cycle with the convex-side limb leading; AIS-A, crossing cycle with the concave-side limb leading; P_H_ = *p*-value for obstacle height; P_V_ = AIS-V vs. Control; P_A_ = AIS-A vs. Control; P_S_ = AIS-A vs. AIS-V; DLS, double-limb support; CSLS, contralateral single-limb support.

*AIS-V or AIS-A significantly different from control.

**Fig 3:**
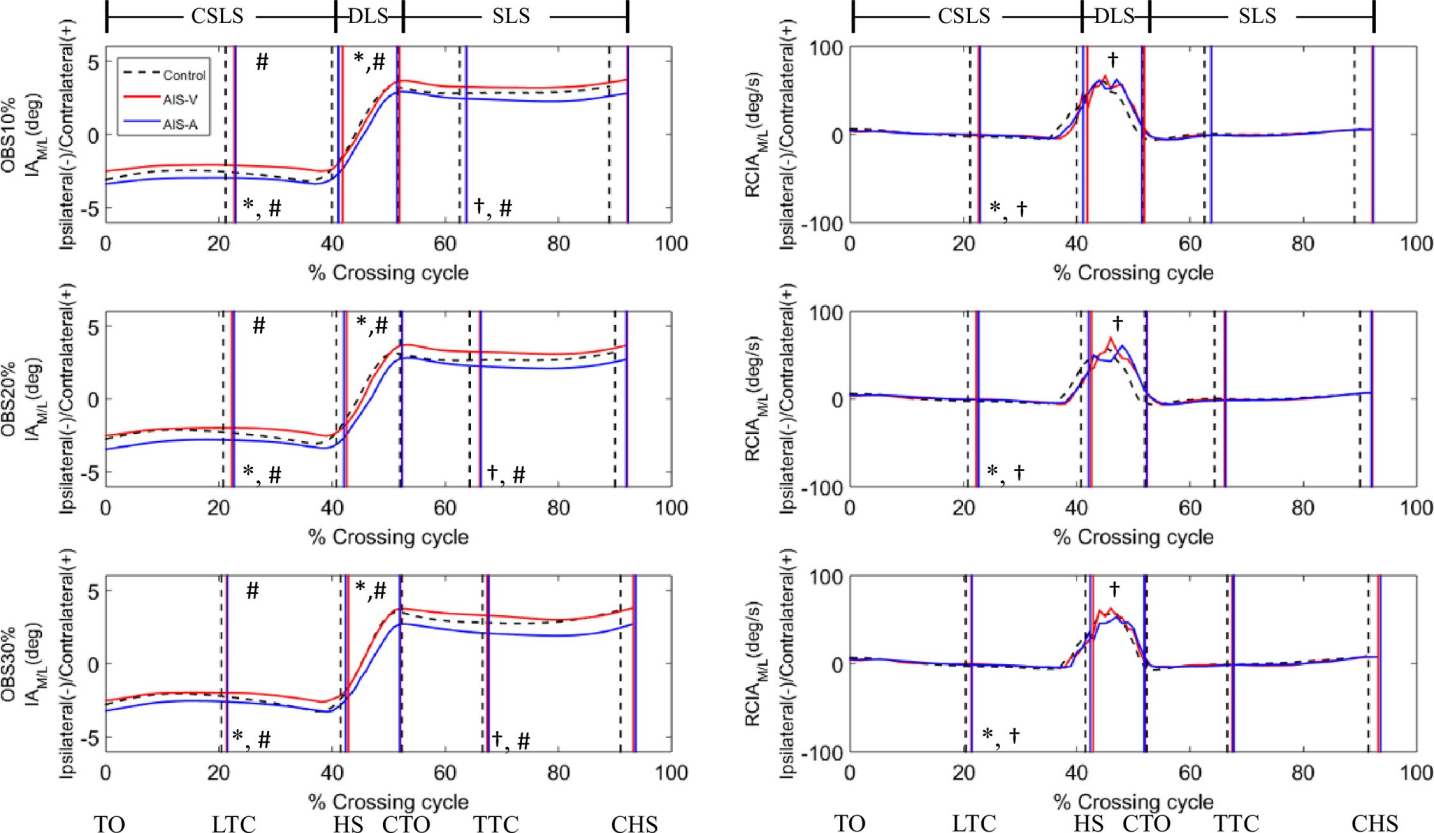
Ensemble-averaged curves of COM-COP inclination angle (IA) (left column), and rate of change of IA (RCIA) (right column) in the frontal plane over the crossing cycle when crossing obstacles of heights of 10%, 20% and 30% the leg length (LL) for convex side, concave side and control, drawn with data points at an interval of 1% crossing cycle. The medial/lateral (M/L) position of the COM and COP were described relative to the line of progression that described the M/L range of motion of the COM during a gait cycle, a positive value being to the side of the contralateral limb. (TO: toe-off of the leading limb; LTC: leading toe above the obstacle; HS: heel-strike of the leading limb; CTO: toe-off of the contralateral limb; TTC: trailing toe above the obstacle; CHS: heel-strike of the contralateral limb; SLS: single-limb support of the leading limb; CSLS: contralateral single-limb support; DLS: double-limb support; *: AIS-A significantly different from Control; †: AIS-V significantly different from Control; #: AIS-A significantly different from AIS-V).

In the frontal plane the AIS-A showed a significantly increased IA magnitude compared to Control while the AIS-V showed unaltered IA when the leading toe was above the obstacle (Figs 2 and 3), with RCIA magnitudes very close to zero for both AIS-A and AIS-V (Table 2). Over the leading swing phase, the AIS-A showed significantly increased IA magnitudes compared to AIS-V. During DLS, while the AIS-V maintained an unaltered mean IA value, the AIS-A significantly altered the IA control to show a negative mean IA when compared to both Control and AIS-V (Table 2). Both AIS-V and AIS-A also showed significantly increased peak values of RCIA but only AIS-V showed significantly increased mean values of RCIA over DLS when compared to Control (Table 3). Over the DLS and CSLS, the AIS-V showed an increased jerk index when compared to Control (Table 4).

With increasing obstacle height, crossing speeds were decreased linearly, while the trailing toe-obstacle distance and leading heel-obstacle distance were found to increase linearly for both groups (Table 1). Linearly decreased DLS time but linearly increased leading swing phase time were found in both groups. In the sagittal plane, both groups linearly decreased anterior IA when the leading toe was above the obstacle (Table 2). When the trailing toe was above the obstacle, increased anterior IA but decreased anterior RCIA were found in both groups (Table 3). Averaged values of the anterior RCIA over the leading swing phase decreased but the peak RCIA was increased with increasing obstacle height (Table 3). No significant obstacle height effects were found in the frontal plane (Tables 2 and 3).

## Discussion

The current study aimed to identify the effects of thoracic AIS on the whole-body balance control during obstacle-crossing in terms of COM-COP IA and RCIA. The results support the hypothesis that, when compared to healthy controls, the patients with AIS crossed obstacles with altered, compromised balance control.

In the sagittal plane, the patients crossed the obstacle with unaltered IA but increased anterior RCIA when crossing, whether with the concave-side or the convex-side leading. Since the COM was ahead of the stance limb when the leading limb was crossing, increased anterior RCIA indicated that the COM was moving away from the stance limb at a faster speed, increasing the difficulty in maintaining a smooth COM motion relative to the stance limb, as indicated by the increased jerk index of IA. Increased anterior RCIA also appeared to contribute to the increased horizontal heel-obstacle distance, requiring greater braking force to slow down the anterior motion of the COM, which appeared to lead to an increased posterior RCIA during DLS for both AIS-A and AIS-V. Rapid changes in RCIA suggest challenges for a smooth transition between swing and stance, as well as for weight transfer during DLS (Table 4).

In the frontal plane, compared to healthy controls the patients appeared to have a compromised balance control with increased IA magnitude but reduced RCIA when the swing toe of the concave-side leading-limb was above the obstacle during crossing, suggesting an increased risk of loss of balance during crossing. Previous studies have shown that the M/L COM stability is an important parameter for distinguishing older people with imbalance, because they showed greater M/L inclination angles [22, 44]. Increased frontal IA, i.e., increased medio-lateral COM-COP separation, increases the difficulty in controlling a smooth and stable COM motion relative to the COP, which was found to lead to loss of balance in elderly fallers, especially under unexpected postural disturbances such as tripping or slipping [22]. For leading with the limb on the convex side, the increased bending of the trunk towards the limb on the concave side [33] may help minimize the separation of the COM and COP, leading to a COM-COP inclination angle similar to that of the healthy controls. These results suggest that the whole-body balance control during obstacle-crossing in patients with Lenke 1 AIS should be monitored for signs of increased risk of loss of balance in the management of such a patient group, especially when crossing obstacles with the limb on the concave side leading.

During the DLS phase after the leading limb had crossed the obstacle, the COM was controlled within a relatively small range while the COP traveled from the trailing to the leading limb during the body weight transfer. Therefore, the smooth motion of the COM relative to the COP in terms of a smooth change in RCIA, and normal or reduced jerk index of IA is an indication of a well-controlled transfer of the body weight while maintaining dynamic balance. In the sagittal plane, both AIS-A and AIS-V increased the peak and mean posterior RCIA, with an increased jerk index of IA during DLS, suggesting a less well-controlled COM-COP motion and an increased effort in transferring the body weight to the leading limb. In the frontal plane during the DLS of AIS-A, the patients appeared to alter the IA control significantly, with prolonged duration of the weight release phase, i.e., the body weight was kept on the trailing limb for longer before the COP traveled to be right below the COM (i.e., zero IA), leading to a negative mean IA during DLS as compared to Control. These results suggest that patients with AIS had a reduced ability in maintaining a smooth and stable transfer of the body weight and COM-COP control from the trailing to the leading limb during obstacle-crossing when leading with the concave-side limb. The vast changes of RCIA around the instances of transfer from swing to DLS, and from DLS to swing, particularly shortly before the beginning and after the end of the DLS when the largest IA magnitudes occurred, are also an indication of an increased difficulty in COM-COP control and thus an increased risk of loss of balance for AIS.

### Study limitations

The current study was the first attempt to identify the effects of AIS on the control of the body’s COM motion relative to the COP in terms of IA and RCIA during obstacle-crossing. The patient group was limited to Lenke 1 thoracic scoliosis without compensatory thoracolumbar curves for better homogeneity of the patient group. Nonetheless, this type of spinal deformity, and thus the trunk shape in AIS, has been shown to affect the motion of the trunk during activities [8, 45, 46], and presumably the COM-COP control. Therefore, further studies are needed to identify how the spinal deformity type and the severity of AIS would affect the COM-COP control during walking and obstacle-crossing. Another factor that may limit the patient’s ability in controlling the trunk and COM-COP motion is muscle strength imbalance [4, 45, 47], which also deserves further investigation.

## Conclusions

The patients with Lenke 1 thoracic AIS were found to cross obstacles with altered, compromised COM-COP control in both sagittal and frontal planes when compared to healthy controls. The patients increased anterior RCIA with an increased jerk index of IA during crossing with either the limb on the concave side or convex side leading, indicating their difficulty in maintaining a smooth COM-COP motion in the sagittal plane. In the frontal plane, the patients adopted a conservative COM-COP control strategy with close-to-zero RCIA during the leading limb crossing. However, their balance control appeared to be compromised with an increased IA magnitude when crossing with the concave-side limb leading. The current results suggest that the thoracic spinal deformity in Lenke 1 AIS affected the whole-body balance control during obstacle-crossing, especially when the concave-side was leading. Thus, balance control should be monitored for signs of increased risk of loss of balance in the management of such patient groups.

## Supporting information

S1 File (XLSX)

## References

[1] Weinstein SL, Dolan LA, Cheng JC, Danielsson A, Morcuende JA. Adolescent idiopathic scoliosis. Lancet. 2008;371:1527–37. 10.1016/S0140-6736(08)60658-3 18456103

[2] Chockalingam N, Bandi S, Rahmatalla A, Dangerfield PH, Ahmed el N. Assessment of the centre of pressure pattern and moments about S2 in scoliotic subjects during normal walking. Scoliosis. 2008;3:10 10.1186/1748-7161-3-10 18699989 PMC2526987

[3] Le Blanc R, Labelle H, Forest F, Poitras B, Rivard CH. Possible relationship between idiopathic scoliosis and morphologic somatotypes in adolescent females. Ann Chir. 1995;49:762–7. 8561432

[4] Cromwell RL, Aadland-Monahan TK, Nelson AT, Stern-Sylvestre SM, Seder B. Sagittal plane analysis of head, neck, and trunk kinematics and electromyographic activity during locomotion. J Orthop Sports Phys Ther. 2001;31:255–62. 10.2519/jospt.2001.31.5.255 11352192

[5] Masso PD, Gorton GE, 3rd. Quantifying changes in standing body segment alignment following spinal instrumentation and fusion in idiopathic scoliosis using an optoelectronic measurement system. Spine (Phila Pa 1976). 2000;25:457–62. 10.1097/00007632-200002150-00011. 10707391

[6] Petersen I, Sahlstrand T, Sellden U. Electroencephalographic investigation of patients with adolescent idiopathic scoliosis. Acta Orthop Scand. 1979;50:283–93. 10.3109/17453677908989769 474099

[7] Sahlstrand T, Petruson B. A study of labyrinthine function in patients with adolescent idiopathic scoliosis. I. An electro-nystagmographic study. Acta Orthop Scand. 1979;50:759–69. 10.3109/17453677908991307 534551

[8] Mahaudens P, Banse X, Mousny M, Detrembleur C. Gait in adolescent idiopathic scoliosis: kinematics and electromyographic analysis. Eur Spine J. 2009;18:512–21. 10.1007/s00586-009-0899-7 19224255 PMC2899459

[9] Barrack RL, Whitecloud TS 3rd, Burke SW, Cook SD, Harding AF. Proprioception in idiopathic scoliosis. Spine (Phila Pa 1976). 1984;9:681–5. 10.1097/00007632-198410000-00005. 6505836

[10] Nault ML, Allard P, Hinse S, Le Blanc R, Caron O, Labelle H, et al Relations between standing stability and body posture parameters in adolescent idiopathic scoliosis. Spine (Phila Pa 1976). 2002;27:1911–7. 10.1097/00007632-200209010-00018. 12221357

[11] Chen PQ, Wang JL, Tsuang YH, Liao TL, Huang PI, Hang YS. The postural stability control and gait pattern of idiopathic scoliosis adolescents. Clin Biomech (Bristol, Avon). 1998;13:S52–S8. 10.1016/S0268-0033(97)00075-2. 11430791

[12] Paul JC, Patel A, Bianco K, Godwin E, Naziri Q, Maier S, et al Gait stability improvement after fusion surgery for adolescent idiopathic scoliosis is influenced by corrective measures in coronal and sagittal planes. Gait Posture. 2014;40:510–5. 10.1016/j.gaitpost.2014.06.006 25023225

[13] Simon AL, Ilharreborde B, Souchet P, Kaufman KR. Dynamic balance assessment during gait in spinal pathologies—a literature review. Orthop Traumatol Surg Res. 2015;101:235–46. 10.1016/j.otsr.2014.11.021 25765946

[14] Schizas CG, Kramers-de Quervain IA, Stussi E, Grob D. Gait asymmetries in patients with idiopathic scoliosis using vertical forces measurement only. Eur Spine J. 1998;7(2):95–8. 10.1007/s005860050037 9629931 PMC3611223

[15] Kramers-de Quervain IA, Muller R, Stacoff A, Grob D, Stussi E. Gait analysis in patients with idiopathic scoliosis. Eur Spine J. 2004;13:449–56. 10.1007/s00586-003-0588-x 15064994 PMC3476595

[16] England SA, Granata KP. The influence of gait speed on local dynamic stability of walking. Gait Posture. 2007;25:172–8. 10.1016/j.gaitpost.2006.03.003 16621565 PMC1785331

[17] Wank V, Frick U, Schmidtbleicher D. Kinematics and electromyography of lower limb muscles in overground and treadmill running. Int J Sports Med. 1998;19:455–61. 10.1055/s-2007-971944 9839841

[18] Sarwahi V, Boachie-Adjei O, Backus SI, Taira G. Characterization of gait function in patients with postsurgical sagittal (flatback) deformity: a prospective study of 21 patients. Spine (Phila Pa 1976). 2002;27:2328–37. 10.1097/01.brs.0000030304.83145.01 12438980

[19] Tinetti ME, Speechley M, Ginter SF. Risk factors for falls among elderly persons living in the community. N Engl J Med. 1988;319:1701–7. 10.1056/NEJM198812293192604 3205267

[20] Karimi MT, Kavyani M, Kamali M. Balance and gait performance of scoliotic subjects: A review of the literature. J Back Musculoskelet Rehabil. 2016;29:403–15. 10.3233/BMR-150641 26519115

[21] Chien HL, Lu TW, Liu MW. Control of the motion of the body's center of mass in relation to the center of pressure during high-heeled gait. Gait Posture. 2013;38:391–6. 10.1016/j.gaitpost.2012.12.015 23337731

[22] Lee HJ, Chou LS. Detection of gait instability using the center of mass and center of pressure inclination angles. Arch Phys Med Rehabil. 2006;87:569–75. 10.1016/j.apmr.2005.11.033 16571399

[23] Pai YC, Patton J. Center of mass velocity-position predictions for balance control. J Biomech. 1997;30:347–54. 10.1016/s0021-9290(96)00165-0 9075002

[24] Huang S-C, Lu T-W, Chen H-L, Wang T-M, Chou L-S. Age and height effects on the center of mass and center of pressure inclination angles during obstacle-crossing2008. 968–75 p. 10.1016/j.medengphy.2007.12.005 18243037

[25] Hong SW, Leu TH, Wang TM, Li JD, Ho WP, Lu TW. Control of body's center of mass motion relative to center of pressure during uphill walking in the elderly. Gait Posture. 2015;42(4):523–8. Epub 2015/09/21. 10.1016/j.gaitpost.2015.08.007 . 26386677

[26] Wang TM, Chen HL, Lu TW. Effects of obstacle height on the control of the body center of mass motion during obstructed gait. Journal of the Chinese Institute of Engineers. 2007;30(3):471–9. 10.1080/02533839.2007.9671275

[27] Hsu WC, Wang TM, Liu MW, Chang CF, Chen HL, Lu TW. Control of Body's Center of Mass Motion During Level Walking and Obstacle-Crossing in Older Patients with Knee Osteoarthritis. Journal of Mechanics. 2011;26(2):229–37. Epub 05/05. 10.1017/S1727719100003087

[28] Hof AL. The 'extrapolated center of mass' concept suggests a simple control of balance in walking. Human Movement Science. 2008;27(1):112–25. 10.1016/j.humov.2007.08.003 WOS:000253579800008. 17935808

[29] Hof AL, Gazendam MG, Sinke WE. The condition for dynamic stability. J Biomech. 2005;38(1):1–8. Epub 2004/11/03. 10.1016/j.jbiomech.2004.03.025 . 15519333

[30] Bruijn SM, van Dieën JH. Control of human gait stability through foot placement. Journal of the Royal Society, Interface. 2018;15(143):20170816 10.1098/rsif.2017.0816 . 29875279 PMC6030625

[31] Sivakumaran S, Schinkel-Ivy A, Masani K, Mansfield A. Relationship between margin of stability and deviations in spatiotemporal gait features in healthy young adults. Hum Mov Sci. 2018;57:366–73. Epub 2017/10/11. 10.1016/j.humov.2017.09.014 28987772 PMC5770210

[32] Hak L, Houdijk H, Beek PJ, van Dieen JH. Steps to take to enhance gait stability: the effect of stride frequency, stride length, and walking speed on local dynamic stability and margins of stability. PLoS One. 2013;8(12):e82842 Epub 2013/12/19. 10.1371/journal.pone.0082842 24349379 PMC3862734

[33] Wu KW, Li JD, Huang HP, Liu YH, Wang TM, Ho YT, et al Bilateral asymmetry in kinematic strategies for obstacle-crossing in adolescents with severe idiopathic thoracic scoliosis. Gait Posture. 2019;71:211–8. 10.1016/j.gaitpost.2019.05.007 31078825

[34] Lenke LG, Betz RR, Haher TR, Lapp MA, Merola AA, Harms J, et al Multisurgeon assessment of surgical decision-making in adolescent idiopathic scoliosis: Curve classification, operative approach, and fusion levels. Spine. 2001;26:2347–53. 10.1097/00007632-200111010-00011 11679820

[35] Erdfelder E, Faul F, Buchner A. GPOWER: A general power analysis program. Behav Res Methods Instrum Comput. 1996;28:1–11. 10.3758/bf03203630.

[36] Chen HL, Lu TW. Comparisons of the joint moments between leading and trailing limb in young adults when stepping over obstacles. Gait Posture. 2006;23:69–77. 10.1016/j.gaitpost.2004.12.001 16311197

[37] Hong SW, Leu TH, Wang TM, Li JD, Ho WP, Lu TW. Control of body's center of mass motion relative to center of pressure during uphill walking in the elderly. Gait Posture. 2015;42:523–8. 10.1016/j.gaitpost.2015.08.007 26386677

[38] Chen SC, Hsieh HJ, Lu TW, Tseng CH. A method for estimating subject-specific body segment inertial parameters in human movement analysis. Gait Posture. 2011;33:695–700. 10.1016/j.gaitpost.2011.03.004 21458993

[39] Woltring HJ. A fortran package for generalized, cross-validatory spline smoothing and differentiation. Advances in Engineering Software and Workstations. 1986;8:104–13. 10.1016/0141-1195(86)90098-7

[40] Young RP, Marteniuk RG. Acquisition of a multi-articular kicking task: Jerk analysis demonstrates movements do not become smoother with learning. Human Movement Science. 1997;16(5):677–701. 10.1016/S0167-9457(97)00010-9.

[41] Rohrer B, Fasoli S, Krebs HI, Hughes R, Volpe B, Frontera WR, et al Movement smoothness changes during stroke recovery. The Journal of neuroscience: the official journal of the Society for Neuroscience. 2002;22(18):8297–304. 10.1523/JNEUROSCI.22-18-08297.2002 . 12223584 PMC6758113

[42] Edelman S, Flash T. A model of handwriting. Biol Cybern. 1987;57(1–2):25–36. Epub 1987/01/01. 10.1007/bf00318713 . 3620543

[43] Hogan N, Sternad D. Sensitivity of smoothness measures to movement duration, amplitude, and arrests. J Mot Behav. 2009;41(6):529–34. 10.3200/35-09-004-RC . 19892658 PMC3470860

[44] Chou LS, Kaufman KR, Hahn ME, Brey RH. Medio-lateral motion of the center of mass during obstacle crossing distinguishes elderly individuals with imbalance. Gait Posture. 2003;18:125–33. 10.1016/s0966-6362(02)00067-x 14667945

[45] Danielsson AJ, Romberg K, Nachemson AL. Spinal range of motion, muscle endurance, and back pain and function at least 20 years after fusion or brace treatment for adolescent idiopathic scoliosis: a case-control study. Spine (Phila Pa 1976). 2006;31:275–83. 10.1097/01.brs.0000197652.52890.71. 16449899

[46] Wu KW, Wang TM, Hu CC, Hong SW, Lee PA, Lu TW. Postural adjustments in adolescent idiopathic thoracic scoliosis during walking. Gait Posture. 2018;68:423–9. 10.1016/j.gaitpost.2018.12.024 30594870

[47] Riddle HFV, Roaf R. Muscle imbalance in the causation of scoliosis. The Lancet. 1955;265:1245–7. 10.1016/S0140-6736(55)91020-5. 14382561

